# Immunogenic, but Not Steady-State, Antigen Presentation Permits Regulatory T-Cells To Control CD8+ T-Cell Effector Differentiation by IL-2 Modulation

**DOI:** 10.1371/journal.pone.0085455

**Published:** 2014-01-13

**Authors:** Alice McNally, Michael McNally, Ryan Galea, Ranjeny Thomas, Raymond J. Steptoe

**Affiliations:** UQ Diamantina Institute, University of Queensland, Brisbane, Australia; Maisonneuve-Rosemont Hospital, Canada

## Abstract

Absorption of IL-2 is one proposed mechanism of CD4+CD25+FoxP3+ regulatory T cell (Treg) suppression. Direct in vivo experimental evidence for this has recently been obtained. While modulation of IL-2 bioavailability controls CD8+ T-cell effector differentiation under strongly immunogenic conditions it is not known whether Treg modulate CD8+ T cell responses through this mechanism under steady-state conditions. Here we assess this using a mouse model in which dendritic cells (DC) are manipulated to present cognate antigen to CD8+ T cells either in the steady-state or after activation. Our observations show that Treg exert a check on expansion and effector differentiation of CD8+ T cells under strongly immunogenic conditions associated with TLR ligand activation of DC, and this is mediated by limiting IL-2 availability. In contrast, when DC remain unactivated, depletion of Treg has little apparent effect on effector differentiation or IL-2 homeostasis. We conclude that while modulation of IL-2 homeostasis is an important mechanism through which Treg control CD8+ effector differentiation under immunogenic conditions, this mechanism plays little role in modulating CD8+ T-cell differentiation under steady-state conditions.

## Introduction

Multiple mechanisms of peripheral tolerance overlap to prevent uncontrolled immune responses to pathogen infection and environmental- or self-antigens. Pathogen-associated signals such as Toll-like receptors (TLR) ligands or other PAMPs can convert DC from steady-state, tolerogenic cells, to ‘licensed’ APC with a strong capacity to induce effector responses. In the absence of infection or inflammation, antigen presentation by steady-state dendritic cells (DC) leads to T cell tolerance where T cells are driven to apoptosis or rendered unresponsive, and this is an important mechanism preventing progression to autoimmune diseases [1], [2]. In addition to APC-mediated control of naive T-cell differentiation, CD4+CD25+FoxP3+ regulatory T cells (Treg) prevent overexuberant T-cell responses by limiting T-cell activation and differentiation in lymphoid tissues and effector function at target sites [3], [4]. Treg also participate in immune regulation and tolerance through mechanisms that include promoting Treg differentiation from naive CD4+ T cells [5] and modulating DC phenotype and function [6]–[8].

Treg exert their influence through diverse immunosuppressive mechanisms (reviewed in [9], [10]) that may differ depending on the context. It has been elegantly shown in a tumour setting, that Treg directly inhibit CD8+ T-cell-mediated cytolysis through mechanisms including TGF-β-dependent inhibition of degranulation [11], [12]. Interestingly, in this setting where antigen-presentation to naive T-cells may occur principally under steady-state or weakly-immunogenic conditions Treg act principally to inhibit effector function whereas priming and effector differentiation appears unaltered [11], [12]. However, in settings that lead to strongly immunogenic priming, such as vaccination, Treg restrain CD8+ T-cell expansion and effector differentiation [13], [14]. Such disparate observations could reflect differences between T-cell activation occurring when DC exist in the steady-state or are strongly activated, for example, by TLR ligands respectively. Alternatively, effector T cells or T cells undergoing effector differentiation may act to promote Treg function which in turn permits control of effector responses. We and others have shown that modulation of IL-2 homeostasis is one key mechanism by which Treg control effector differentiation of CD8+ T cells whereby uptake of IL-2 by Treg both limits CD8+ effector differentiation and promotes Treg expansion [14]–[17]. It is clear that this mechanism is a powerful controller of CD8+ T cells undergoing effector differentiation but it remains unclear whether this contributes to control of the CD8+ T cells responding to steady-state antigen presentation.

Here we determined the role of Treg in modulating CD8+ T cells responses in a murine model of DC antigen presentation under conditions promoting either tolerance or immunity. In steady-state conditions, expansion and transient development of effector function of CD8+ T cells activated by steady-state DC was unaltered by depletion of Treg by αCD25 administration. In contrast, under immunogenic conditions when DC were ‘licensed’ by TLR stimulation, depletion of Treg increased CD8+ effector differentiation. Blockade of IL-2 in vivo did not affect CD8+ responses under conditions of steady-state antigen presentation, but reversed the additional T cell expansion induced by Treg depletion under immunogenic conditions. Together the data indicate that control of IL-2 homeostasis by Treg modulates immunogenic but not steady-state T-cell responses.

## Materials and Methods

### Ethics Statement

This study was carried out in accordance with the guidelines of the Australian Code of Practice for the Care and Use of Animals for Scientific Purposes. All experiments were approved by The University of Queensland Animal Ethics Committee (projects 251/08, 185/11).

### Mice

Mice were from the Animal Resources Centre (Perth, WA, Australia) or bred and maintained at the Biological Research Facility (Woolloongabba, QLD, Australia). OT-I mice carrying a transgenic TCR for H-2K^b^/OVA_257–264_ [18] were bred with C57BL/6.SJL*ptprca* mice to generate CD45.1+ OT-I mice. CD11c.OVA mice have been described [19].

### Antibodies and in vitro Analyses

mAb for cytometry were from Biolegend (San Diego, CA, USA) or BD (San Jose, CA). αCD25 (PC61) and αphytochrome (*Avena sativa*) (MAC49) were purified from hybridoma supernatants in house and αIL-2 (JES6-1, S4B6) was purchased from BioXcell (Lebanon, NH, USA). FoxP3 staining reagents and ELISA mAb were from eBioscience (San Diego, CA, USA). For DC phenotyping, spleens were collected, digested with collagenase/DNAse and cells collected for flow cytometric analysis [20]. For Treg suppression assays CD4+CD25− T cells and CD4+CD25+ Treg were enriched using magnetic separation (CD4^+^CD25^+^ Regulatory T Cell Isolation Kit, Miltenyi Biotec Australia) and CD11c+ DC collected by magnetic separation (Miltenyi Biotec Australia, Macquarie Park, NSW, Australia) according to manufacturer’s recommendations. Treg suppression assays were established using CD4+CD25− responders, CD11c+ DC and titrated numbers of CD4+CD25+ Treg as described [21]. For flow cytometry, cells were stained and analyzed as described previously [19] using a flow bead based counting assay [22] where indicated. Flow cytometric data were collected using BD FACSCalibur or BD FACSCanto cytometers and analysed using CellQuest or Diva software (BD) and displays for publication prepared using or FlowJo (TreeStar).

### *In vivo* Analyses

CD25^+^ cells were depleted using αCD25 (PC61, 1 mg) administration every 3 days. Controls were treated identically with isotype-matched αphytochrome mAb (MAC-4). For in vivo IL-2 blockade αIL-2 mAb (JES6-1, S4B6 50) were mixed (200 ug of each) and injected i.p. daily as described [14]. For DC activation, 10 nmol CpG 1668 (Geneworks, Australia) was injected i.v. at the time of OT-I transfer. When DC phenotype was analysed CpG was injected 18 hours before spleen collection. For OT-I transfer, brachial, axillary, inguinal and mesenteric LN were collected from CD45.1+ OT-I mice and CD8+ T- cells purified (>90% CD8+, <3% CD4+) by negative selection using magnetic beads (Miltenyi Biotec, Auburn, CA) and 2×10^6^ cells injected i.v. In a small number of experiments OT-I donor mice were treated with αCD25 and bulk LN cells containing 2×10^6^ CD8+ (OT-I) T cells transferred. Serum was prepared from blood obtained by cardiac puncture and ELISA performed using JES6-1A12/JES6-5H4 (capture/detection) using a standard protocols.

### Statistical Analyses

Students *t*-test was used to compare means and one way ANOVA followed by Newman-Keuls post test (GraphPad 5, San Diego, CA) for multiple pairwise comparisons.

## Results

### Treg Control Expansion and Contraction of CD8+ T cell Responses when Antigen is Presented by Activated, but not Steady-state, DC

**Figure 1:**
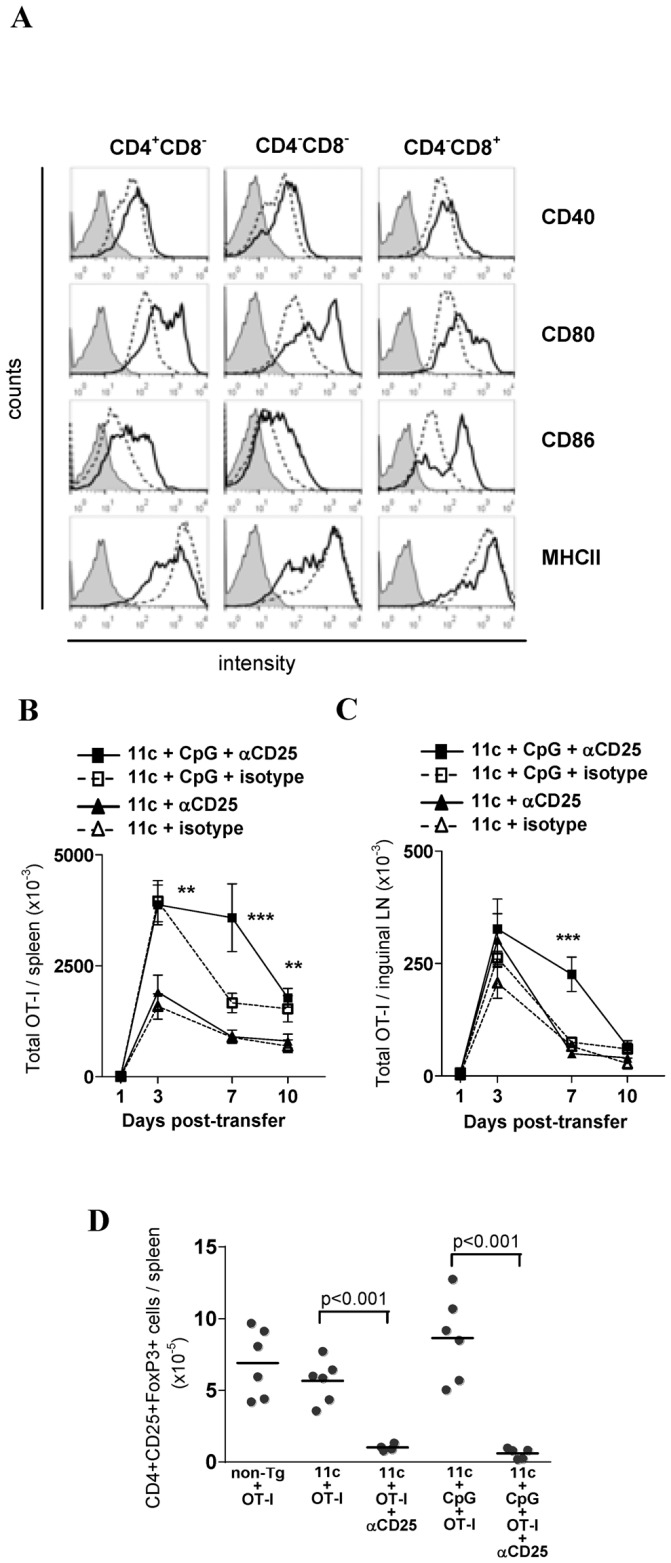
Treg limit CD8+ T-cell expansion when antigen is presented by activated but not steady-state DC. A) C57Bl/6 mice were administered 10 nmol CpG i.v. or left untreated. Eighteen hours later spleens were harvested, digested and DC subsets analysed by flow cytometry. Plots are representative of one mouse of 2–3 analysed in each of 2 separate experiments. B-D) 11c.OVA mice were injected with αCD25 or isotype-control mAb and 3 days later 2×10^6^ CD45.1+ OT-I T-cells were transferred with or without CpG administration at the time of transfer. Additional αCD25 or isotype-control mAb (iso) were administered every 3 days after the initial injection. At the times shown the total number of OT-I (CD45.1^+^CD8^+^Vα2^+^) T cells in the inguinal LN (B) or spleen (C) or at day 7 the number of CD4+CD25+FoxP3+ Treg in spleen were determined using a FACS-based assay. Data were pooled from more than 2 independent experiments (B,C; n = 5–12/group except day 1 where n = 2/group) and bars show mean ± SEM or 2 experiments (D; n = 5 or 6). (B)***: 11c+CpG+αCD25 is greater than others at day 7 (p<0.001) (C) **: 11c+CpG+αCD25 and 11c+CpG are greater than others at day 3 and day 10 (p<0.01), ***: 11c+CpG+αCD25 is greater than others at day 7 (p<0.001).

Here we set out to test the Treg control of CD8+ T-cell responses under highly- or poorly-immunogenic conditions. To achieve this, we used 11c.OVA mouse model of steady-state DC antigen presentation we have described previously [19]. In this model, in the absence of DC activation, DC present OVA-derived peptides in the steady-state leading to abortive activation of OVA-specific T cells and CD8+ T-cell tolerance induction through deletion and induction of unresponsiveness. In order to compare CD8+ T-cell activation by steady-state and activated DC, we first established suitable experimental conditions for DC activation by comparing DC activation with various TLR ligands. In response to systemic CpG1668 expression of the classic activation markers CD40, CD80, CD86 and MHC class II by each of the major conventional DC subsets in spleen was upregulated (Fig. 1A).

To test the effect of Treg depletion on OVA-specific CD8+ T-cell accumulation in response to antigen presentation by activated or steady-state DC, CD8+ TCR transgenic (OT-I) T cells were transferred to 11c.OVA mice treated with or without CpG and Treg depletion. In the absence of CpG treatment OT-I T cells transiently expanded in number, peaking 3 days post-transfer in spleen and LN (Fig. 1B, C). This was followed by contraction of the OT-I population (Fig. 1B, C). Typically, residual OVA-specific T cells that remain after population contraction are unresponsive to further antigen stimulation [19]. When DC were activated by CpG administration, accumulation of OT-I T cells was significantly increased in spleen (Fig. 1B) at days 3, 7 and 10 after transfer. Intriguingly, CpG administration slightly increase OT-I accumulation in iLN at day3 (Fig. 1C) but this was not significant. This may be due to the greater activation of DC in spleen that in LN results from i.v. injected TLR ligands [23], [24] and our unpublished observations that i.v. CpG leads to a higher degree of DC activation in spleen than LN.

Depletion of Treg by administration of αCD25 antibody did not alter the scale and kinetics of OT-I expansion nor the onset of population contraction in 11c.OVA mice (Fig. 1B, C). Depletion of CD4+CD25+ Treg typically exceeded 95% at the commencement of the experiments (5.93±0.8 vs 0.23±0.15×10^5^ CD4+/CD25+/FoxP3+ per spleen in undepleted and depleted non-Tg mice respectively) and this was sustained throughout the experiments (e.g Fig. 1D). This indicates that Treg exerted little if any influence over OT-I T cell activation in the absence of DC activation. In 11c.OVA mice administered CpG and OT-I T cells, Treg depletion sustained the accumulation of OT-I T cells and a delayed onset of deletion-induced contraction in both spleen and LN relative to mice with intact Treg populations. The number of OVA-specific CD8+ T cells remained substantially greater in both LN and spleen of Treg-depleted mice 7 days after transfer (Fig. 1B, C). However, the prolonged accumulation of OT-I T cells was transient and by 10 days after transfer, OT-I T cells were present in similar numbers regardless of whether Treg were depleted or not. A similar increased accumulation of differentiating CD8+ effector T cells has been shown in immunised Treg-depleted mice [14].

**Figure 2:**
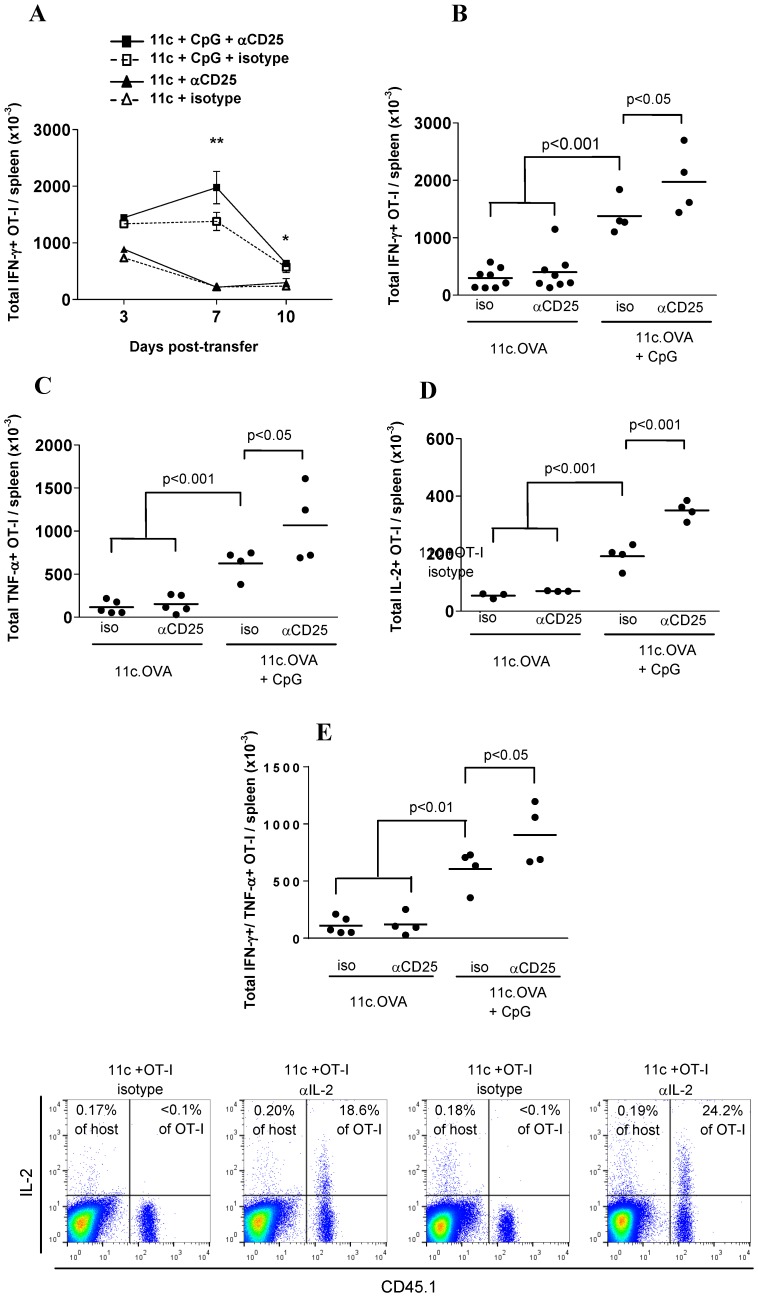
Treg limit effector differentiation of CD8+ T cells activated under immunogenic conditions. A-D) 11c.OVA mice were injected with αCD25 or isotype-control mAb and 3 days later 2×10^6^ CD45.1+ OT-I T-cells transferred with or without CpG administration. Additional αCD25 or isotype-control mAb (iso) was administered every 3 days after the initial injection. At the times shown (A) or 7 days after OT-I transfer (B-F) the total number of OT-I (CD45.1^+^CD8^+^ Vα2^+^ ) T cells in spleen producing IFN-γ, TNF-α, IL-2 or co-producing IFN-γ and TNF-α was determined by ICC and a FACS-based counting assay. IL-2 production 7 days after transfer in OVA_257–264_ restimulated cultures is displayed for host (CD45.-ve left quadrants) or OT-I (CD45.1+ve, right quadrants) cells. Numbers depict the proportion of host (CD45.1-ve) or OT-I cells (CD45.1+ve) staining positively for IL-2. Data shown are (A) mean ± SEM (n = 4) or (B-E) the actual cytokine producing cell numbers for each individual cytokine from individual mice pooled from at least two independent experiments. (A) **: 11c+CpG+αCD25 is greater than 11c+CpG+iso (p<0.05) and greater than 11c+αCD25 and 11c+iso (p<0.01), *11c+CpG+aCD25 and 11c+CpG+iso are greater than others (p<0.05) (one-way ANOVA, Newman-Keuls post-test. GraphPad Prism V5.03). (F) Data are representative of analyses shown in D-E.

Next, to test whether the accumulating OT-I T cells displayed effector function, production of the effector cytokine IFN-γ was measured. Accumulation of IFN-γ-producing OT-I T cells in spleen across days 3, 7 and 10 after transfer was increased by CpG administration (Fig. 2A). Treg depletion further increased accumulation of IFN-γ-producing OT-I T cells in spleen of CpG-treated 11c.OVA mice 7 days after transfer (Fig. 2A, B). Polyfunctional CD8+ T cells, those simultaneously producing multiple cytokines, are considered more potent effectors than those producing IFN-γ alone. Depletion of Treg also increased the accumulation of TNF-α and IL-2 producing OT-I T cells over that in 11c.OVA recipients treated with CpG alone (Fig. 2C, D). Co-staining showed that many of these cells coexpressed IFN-γ and TNF-α (Fig. 2E). Notably, depletion of Treg alone in the absence of CpG had little effect on accumulation of cytokine producing OT-I T cells (Fig. 2B–E). Intracellular cytokine staining was used to determine the predominant cellular source of IL-2. Under both steady-state and immunogenic conditions in spleen, specific IL-2 staining was restricted principally to CD45.1+ve OT-I T cells (Fig. 2F, right quadrants of each plot) whereas IL-2 staining was similar to background isotype control staining in CD45.1-ve cells (Fig. 2F, left hand quadrants in each plot). The proportion of OT-I producing IL-2 was increased in CpG treated 11c.OVA mice relative to controls (Fig. 2F, proportion of IL-2+ve for non-OT-I and OT-I cells shown in cytometry plots). Overall, these data indicate accumulation of differentiating OT-I effector cells was increased by Treg depletion under immunogenic conditions.

**Figure 3:**
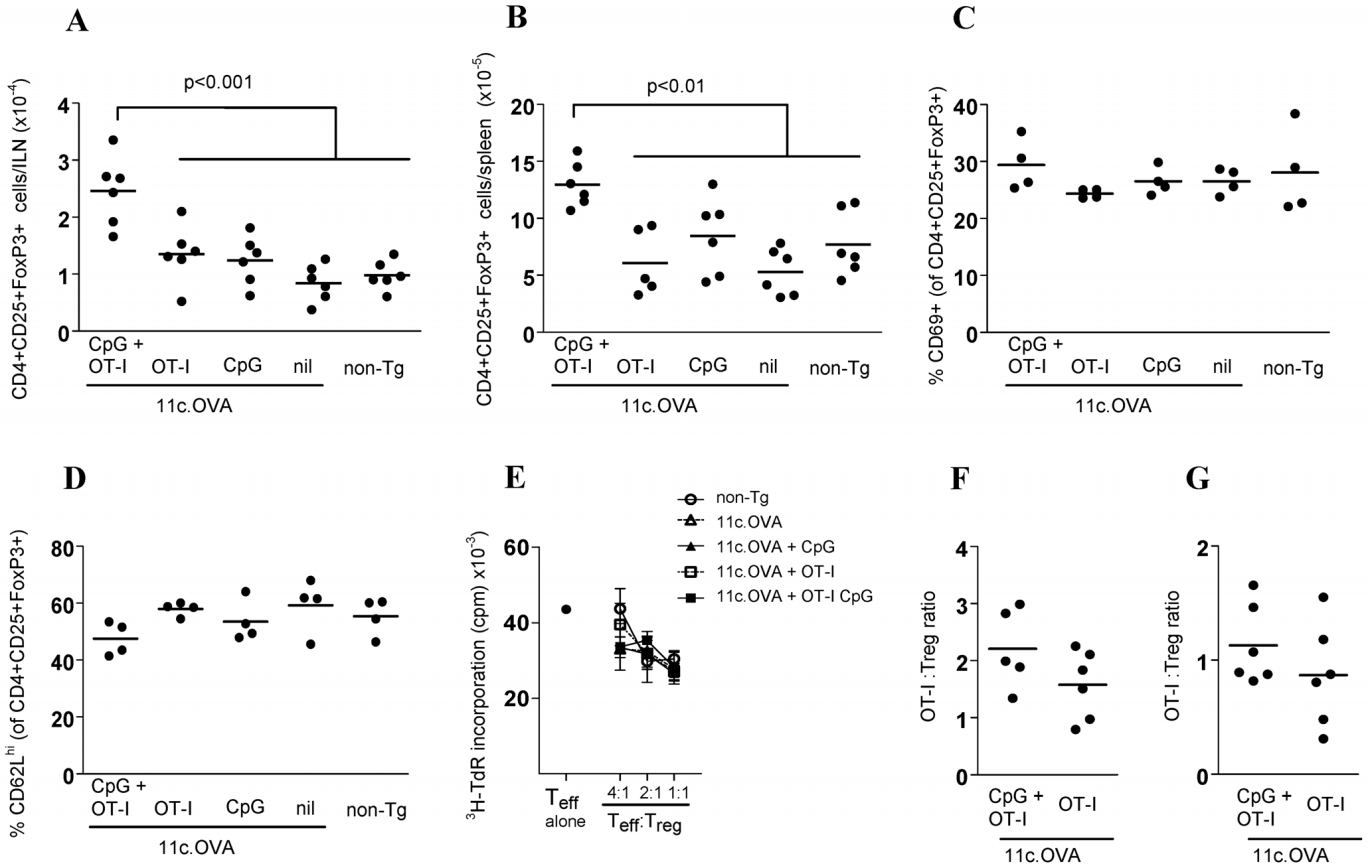
nTreg expand but suppressive activity is similar between immunogenic and steady state conditions. A–D) Non-transgenic (non-Tg) and 11c.OVA mice were administered CpG or not and CD45.1+ OT-I T cells (2×10^6^) mice were transferred as indicated. Five days later, inguinal LN (ILN) and spleens were collected and (A,B) the total number of CD4^+^CD25^+^Foxp3^+^ cells and their expression of (C) CD69 and (D) CD62L determined by flow cytometry. E) Non-transgenic (non-Tg) and 11c.OVA mice were administered CpG or not and CD45.1+ OT-I T cells (2×10^6^) were transferred as indicated. Five days later, spleens were collected and CD4+CD25+ Treg were isolated and added to suppression assays as described in Materials and Methods. F, G) The total number of OT-I T cells was determined as described in Figure 1 and the CD4+CD25+FoxP3+ Treg: OT-I ratio calculated for spleen (F) and ILN (G).

The data indicate that Treg provide a strong modulatory effect on CD8+ T cell responses initiated under immunogenic but not steady-state conditions. This suggests that activation of CD8+ effector differentiation licenses Treg for suppression either by increasing their number and/or function or by providing an opportunity for Treg to exert suppressive mechanisms that are ineffective under poorly-immunogenic conditions. As the presence of a potent immune response can lead to expansion of Treg populations [14], [25] we tested whether Treg were expanded as a consequence of OT-I activation by CpG-activated DC. The total number of Treg recovered from the iLN and spleen of 11c.OVA mice treated with CpG and/or OT-I transfer and untreated controls was compared. Five days after OT-I transfer the total number of Treg was significantly increased in ILN and spleen of 11c.OVA recipients of OT-I T cells and CpG relative to recipients of OT-I T cells without CpG or CpG without OT-I T cells. Therefore, presentation of OVA to OT-I T cells by CpG-activated, but not steady-state DC led to a systemic increase in Treg number. We next determined whether this increase reflected a change in the activation or functional state of Treg under immunogenic conditions. Expression of CD69 and CD62L, surface markers that correlate with Treg activation, did not differ between groups (Fig. 3C, D). In a conventional in vitro suppression assay, there was no substantial difference in the suppressive capacity of Treg from steady-state non-transgenic or 11c.OVA mice and 11c.OVA mice with or without CpG treatment or OT-I transfer. There was no significant change in the ratio of OT-I to Treg in OT-I recipient spleens (Fig. 3F) or ILN (Fig. 3G) whether DC were activated (+CpG) or not (no CpG). Collectively, these data indicate that differential regulation of CD8+ effector differentiation between conditions where DC are activated (immunogenic conditions) or not (tolerogenic conditions) is unlikely to be due to major differences in the ratio of Treg to differentiating effectors, activation state, or function although a role for an increase in the overall number of Treg cannot be ruled out.

### Treg Control of IL-2 Homeostasis is a Key Regulator of Immunogenic, but not Steady-state, CD8+ T-cell Responses

**Figure 4:**
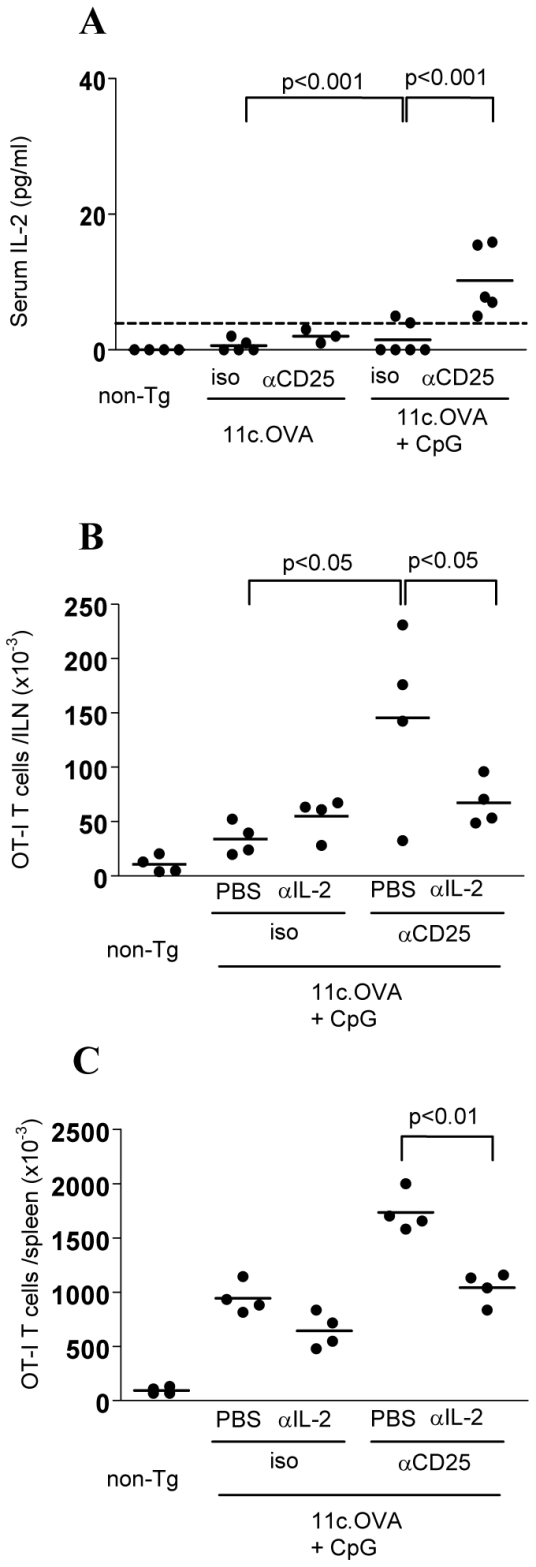
Treg control effector differentiation by modulating IL-2 homeostasis under immunogenic, but not steady state conditions. A) Non-transgenic (non-Tg) and 11c.OVA mice were injected with αCD25 or isotype-control mAb (iso) and 3 days later 2×10^6^ CD45.1+ OT-I T-cells transferred with or without CpG administration. Additional αCD25 or isotype-control mAb were administered every 3 days after the initial injection. 7 days after OT-I transfer, blood was collected and serum prepared. Serum IL-2 concentration was determined by ELISA. Dotted line indicates detection limit. B-D) Non-transgenic (non-Tg) and 11c.OVA mice were injected with αCD25 or isotype-control mAb (iso) and 3 days later 2×10^6^ CD45.1+ OT-I T-cells transferred with or without CpG administration as indicated. αCD25 or isotype-control mAb was administered every 3 days throughout the experiment. In addition, mice were administered either PBS or IL-2 blocking antibodies (JES6.1/S4B6 combined) daily. Seven days after OT-I transfer, inguinal LN (ILN) and spleens were collected and the total number of OT-I (CD45.1+CD8+Vα2+) T cells and the total number of IL-2-producing OT-I T cells determined using a FACS-based counting assay and ICC. Data portray values of individual mice pooled from at least 2 independent experiments.

Modulation of IL-2 homeostasis is an important mechanism by which Treg modulate CD8+ effector differentiation under strongly immunogenic conditions. IL-2 is normally regulated tightly and present at only very low or undetectable levels in serum. Consistent with this, IL-2 was undetectable in the serum of non-transgenic and 11c.OVA recipients of OT-I T cells (Fig. 4A). Previously we have shown that depletion of Treg permits systemic accumulation of IL-2 under immunogenic conditions. Here, however, in the absence of DC activation IL-2 was undetectable or present at low levels in serum with or without depletion of Treg with αCD25 antibody (Fig. 4A). As CD25+ Treg are a key ‘sink’ for IL-2, this data indicates that IL-2 production is minimal under steady-state conditions. Depletion of Treg, however, significantly increased serum IL-2 levels in CpG-treated OT-I recipients (Fig. 4A). This indicated that under CpG-activated conditions substantial IL-2 was produced by activated OT-I T cells as indicated in Figure 2F and, that when present, Treg contributed to absorption of this IL-2. To determine whether the IL-2 that accumulated in CpG-treated OT-I recipients in the absence of Treg might be responsible for increased CD8+ effector accumulation under these conditions, we blocked IL-2 from binding its receptor using a combination of αIL-2 mAb [14]. IL-2 blockade reversed the accumulation of OT-I T cells in LN (Fig. 4B) and spleen (Fig. 4C) resulting from Treg depletion indicating that the sustained accumulation of differentiating CD8+ OT-I effectors was mediated by IL-2 available as a consequence of Treg depletion.

## Discussion

Treg are critical controllers of adaptive immune responses and limit the exuberance of T-cell responses to prevent pathology. As we and others have shown [14], [15] regulation of IL-2 availability by Treg is an important controller of CD8+ effector accumulation and differentiation under highly immunogenic conditions. However, the specific mechanisms by which Treg control CD8+ T-cell responses may differ between immunogenic and non-immunogenic conditions. In a model of constitutive DC antigen expression we demonstrate that modulation of IL-2 availability by Treg is an active control mechanism during strongly-immunogenic responses but not under poorly–immunogenic conditions.

Previous reports suggest that under weakly immunogenic or tolerogenic conditions Treg control over CD8+ effector differentiation may be minimal whereas potent Treg inhibition of CD8+ T-cell effector differentiation is observed when strong effector responses are generated. For instance in a tumour setting, where antigen presentation occurs in the absence of strong innate activating signals, Treg can directly inhibit CD8+ T-cell-mediated cytolysis through TGF-β-dependent inhibition of degranulation [11], [12] but appear not to modulate effector differentiation. In contrast to this, in settings that lead to strong priming, such as vaccination or viral infection, Treg restrain CD8+ T cell expansion and effector differentiation [13], [26]. These disparate observations could reflect antigen presentation by ‘resting’ (tolerogenic) or ‘activated’ (immunogenic) DC respectively. It is also possible that Treg suppressive function is facilitated by T-cell effector differentiation, for example, in response to strongly immunogenic T-cell priming and that only under these conditions does Treg suppression of the response become readily apparent. We manipulated a previously described model of constitutive DC antigen presentation so that steady-state and CpG-activated DC could be compared directly without the introduction of artefacts associated with immunisations such as antigen-processing and trafficking. Our data clearly demonstrate that Treg exert a check on the expansion and effector differentiation of CD8+ T cells under strongly immunogenic conditions associated with DC activation by a TLR ligand. Depletion of CD4+CD25+ Treg under these conditions was associated with increased levels of IL-2 and blockade of IL-2 reversed increased effector differentiation, indicating a direct role for IL-2 in driving effector differentiation. Together this showed Treg were exerting control over CD8+ effector differentiation through control of IL-2 availability. In contrast, when DC remained unactivated, depletion of Treg had no apparent effect on effector differentiation or IL-2 production/accumulation. Therefore, Treg control CD8+ effector differentiation by modulation of IL-2 availability when DC are ‘activated’ but Treg play little or no role in regulating CD8+ effector differentiation under resting conditions. Our data suggest that this context-dependent difference is most likely due to the limited production of IL-2 by CD8+ T cells activated by resting DC.

Treg can control T-cell activation through inhibition of DC function and this may be mediated in part through CTLA4 on Treg [27]. Depletion of Treg can promote DC activation and this could be mediated either directly in the short term, or more chronically via activation of autoreactive T cells [6], [7], [28]. It is plausible that such mechanisms act to promote CD8+ effector differentiation in the absence of Treg. However, our own observations showed no significant change in DC activation markers after anti-CD25 administration in these studies (unpublished observations) suggesting this mechanism played little role here. Depletion of FoxP3+ Treg rather than CD25+ cells might be required and differences resulting from Treg depletion by FoxP3-directed or CD25-directed means cannot be ruled out. However, it is also possible that activation of DC by TLR ligands obviates Treg effects on DC. Certainly, ligation of CD40 can overcome Treg control of immature DC activation and function [29]. However, in either case, DC activation by, for example, TLR ligands may be required for effective CD8+ T-cell effector differentiation as depletion of Treg does not reverse the requirement for costimulation via CD28 for CD8+ T-cell activation [30]. Interestingly, under some conditions, TLR stimulation could not only activate DC to promote effector T-cell priming, but also act to inhibit Treg function thereby further promoting T-cell effector differentiation by the co-incidentally activated DC [31]–[33] this is likely counterbalanced by DC activation acting to increase IL-2 production by differentiating CD8+ effectors. Overall, the data suggest that the ultimate effect on development of CD8+ effector T cell responses of DC activation is influenced not only by DC activation, but also by the balance of factors acting upon Treg.

**Figure 5:**
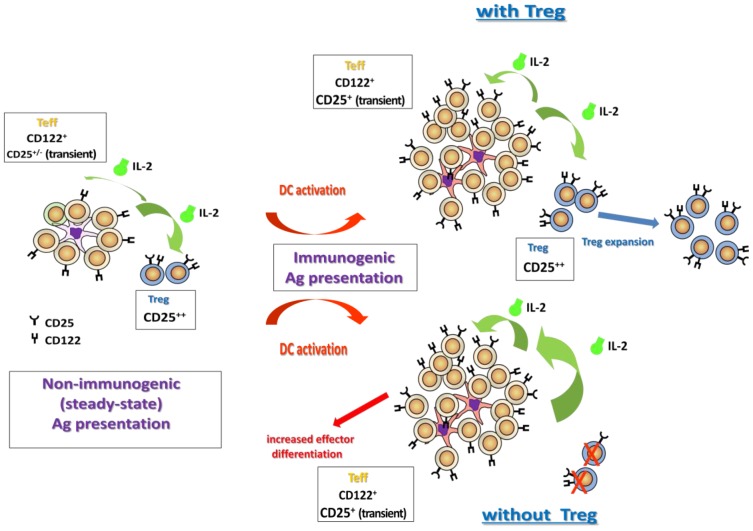
Model of IL-2 modulation in the co-regulation of CD8+ T effector and Treg homeostasis. Under steady-state conditions (LH panel), IL-2 uptake is dominated by Treg expressing the trimeric αβγ high affinity IL-2 receptor (large green arrow, left-hand side). This serves to tightly regulate available IL-2 to low levels. CD8+ T-cell activation under steady-state or poorly immunogenic conditions induces little IL-2 production and IL-2 produced is readily absorbed by CD25+ Treg (large green arrow, left-hand side) to maintain Treg and to limit CD8+ effector differentiation. When DC are activated (red arrow), CD8 expansion and effector differentiation is promoted, which in turn promotes production of IL-2. Under these conditions, increased IL-2 production and transient high level CD25 expression by differentiating CD8+ T cells has the potential to shift IL-2 equilibrium with the antigen-activated differentiating CD8+ population now able to successfully compete with Treg for available IL-2 (Intermediate green arrow upper right-hand side). Abundant IL-2 also boosts CD25 expression by CD8+ T cells. Treg still limit IL-2 availability under these conditions, possibly because they expand or upregulate CD25 in response to the more abundant IL-2. When Treg are depleted, IL-2 is available in excess and substantially promotes CD8+ effector differentiation (large green arrow right-hand side). Abundant IL-2 can signal CD8+ T cells that express the low affinity IL-2R as CD25 expression wanes on post-activated CD8+ T cells. Size of the lettering represents relative expression of IL-2R subunits.

Our data support a suggested model [16], [34], [35] where the effects of IL-2 are determined by relative expression of the high and low affinity IL-2 receptors. As the αβγ(CD25/CD122/CD132) high affinity IL-2 receptor exhibits an IL-2 affinity approx 100-fold higher than that of the low affinity dimeric βγ (CD122/CD132) receptor, equilibrium strongly favours IL-2 binding and uptake by cells bearing the high affinity receptor. Under steady-state conditions, uptake of the low levels of available IL-2 is dominated by CD25+ Treg expressing the trimeric αβγ high affinity IL-2 receptor. This serves to tightly regulate available IL-2 to low levels (e.g. see Fig. 5). CD8+ T-cell activation under steady-state conditions induces little IL-2 production and any IL-2 produced is readily absorbed by CD25+ Treg. Under these conditions IL-2 cannot feed-forward to antigen-activated T cells and effector differentiation remains limited (Fig. 5). Because of the small amounts of IL-2 produced this has little impact on CD25+ Treg homeostasis. However, when DC are activated, CD8 expansion and effector differentiation and IL-2 production is promoted. Under these conditions, transient high level CD25 expression by differentiating CD8+ effector T cells has the potential to shift IL-2 equilibrium with the antigen-activated CD8+ population now able to successfully compete with Treg for available IL-2. However, our data indicate that Treg still limit IL-2 availability under these conditions, possibly because they expand in response to the more abundant IL-2. This, in conjunction with diminishing expression of CD25 by CD8+ [14], will limit the effect of IL-2 on CD8+ T cells and the response begins to wane, controlled by the Treg. Available IL-2 can then be absorbed by Treg as they now have an advantage over the low-affinity IL-2R expressing CD8+ T cells at this stage of the response. When Treg are depleted, excess IL-2 is available and spills into the circulation and substantially promotes effector differentiation. Notably, under these circumstances even CD8+ T cells expressing only the low-affinity βγ IL-2R can be signalled by IL-2 [14]. Homeostasis of IL-2 is therefore determined by a highly dynamic balance between the ratios of Treg and Teff and their relative expression of IL-2R subunits, but non-cognate IL-2R expressing cells may also contribute to this (Fig. 5). Interestingly, CD25 expression by differentiating CD8 is controlled by the presence of antigen and DC activation levels (reviewed in [36]). While we saw no difference in expression of CD25 by OT-I in CpG-treated versus untreated 11c.OVA recipients, failure of CD8+ T cells to express CD25 has been reported in some tolerogenic settings [37], [38]. Reduced CD25 expression under such circumstances may be crucial to limit effector differentiation of CD8+ T cells during tolerance induction. In fact, the level of CD25 expression may be correlated with the extent of effector function exhibited by CD8+ T-cells undergoing tolerance induction. During tolerance induction induced by DC constitutively-expressing antigen we have observed CD25 expression by OT-I T cells during tolerance induction and this coincides with some effector function [19]. In other settings where CD25 fails to be expressed by CD8+ T cells during tolerance induction little effector function is observed [37], [38]. Conversely, under conditions of sustained IL2R signalling [39] or DC activation in the presence of pathogen, IL-2 provides a mechanism to promote CD8 effector differentiation, and amplify CD8+ effector T-cell responses. Overall, IL-2 availability and CD25 expression act as a rheostat for CD8+ effector differentiation, but the scale of the IL-2/CD25-mediated effects are also modulated by Treg.

Under steady-state conditions it is thought that IL-2 is produced primarily by activated CD4+ helper T cells and that these cells therefore serve to maintain CD4+25+ Treg [40], [41]. During strongly immunogenic conditions CD4+ T cells have also been considered the principal source of IL-2 to maintain Treg (reviewed in [35], [36]) even though both CD4+ and CD8+ T cells can produce IL-2. Because 11c.OVA mice are devoid of endogenous OVA-specific T cells, T-cell responses here are restricted to activation of the transferred OVA-specific CD8+ T cells. At least under these circumstances, IL-2 required to expand Treg can be derived from differentiating CD8+ effectors rather than necessarily from bystander activation of CD4+ T cells. However, inclusion of IL_2-deficient CD8+ T cells in future studies would provide definitive demonstration of this.

Our observations differ from the role of Treg reported in another model of DC-induced tolerance where diphtheria toxin (DT)-induced Treg depletion inhibited induction of CD8+ T cell tolerance by steady-state DC. In the latter model, cognate antigen is transiently expressed (induced by a single injection of tamoxifen) and only in approximately 5% of DC [7]. This differs substantially from the model reported here where antigen is constitutively expressed and presented by all DC. These alternate experimental settings may reveal different requirements for Treg under these specific conditions or, alternatively, differences could be due to the procedures used for Treg depletion. In the current study only CD4+CD25+ Treg were depleted and this may be insufficient to release DC from control of (residual) CD4+CD25−FoxP3+ Treg that remain after αCD25-mediated depletion. CD4+CD25−FoxP3+ Treg have been reported to exert suppressive activity in other settings. In contrast, depletion of both CD25+FoxP3+ and CD25− FoxP3+ Treg promotes DC ‘activation’ spontaneously or in response to interaction with activated self-reactive T cells [6]–[8]. Under the latter circumstances, Treg may regulate DC activation rather than the modulation of IL-2 described here that becomes crucial under strongly immunogenic conditions. Overall, in combination with those of others, our observations strongly support a conclusion that different mechanisms of Treg suppression dominate in controlling CD8+ T-cell responses under conditions where DC exist either as ‘steady-state’ or ‘activated’ antigen-presenting cells.

## References

[1] Luckashenak N, Schroeder S, Endt K, Schmidt D, Mahnke K, et al (2008) Constitutive crosspresentation of tissue antigens by dendritic cells controls CD8+ T cell tolerance in vivo. Immunity 28: 521–532. 18387832 10.1016/j.immuni.2008.02.018

[2] Ohnmacht C, Pullner A, King SB, Drexler I, Meier S, et al (2009) Constitutive ablation of dendritic cells breaks self-tolerance of CD4 T cells and results in spontaneous fatal autoimmunity. J Exp Med 206: 549–559. 19237601 10.1084/jem.20082394 PMC2699126

[3] Tang Q, Adams JY, Tooley AJ, Bi M, Fife BT, et al (2005) Visualizing regulatory T cell control of autoimmune responses in nonobese diabetic mice. Nat Immunol 7: 83–92. 16311599 10.1038/ni1289 PMC3057888

[4] Chen Z, Herman AE, Matos M, Mathis D, Benoist C (2005) Where CD4+CD25+ T reg cells impinge on autoimmune diabetes. J Exp Med 202: 1387–1397. 16301745 10.1084/jem.20051409 PMC2212985

[5] Jonuleit H, Schmitt E, Kakirman H, Stassen M, Knop J, et al (2002) Infectious tolerance: human CD25(+) regulatory T cells convey suppressor activity to conventional CD4(+) T helper cells. J Exp Med 196: 255–260. 12119350 10.1084/jem.20020394 PMC2193929

[6] Kim JM, Rasmussen JP, Rudensky AY (2007) Regulatory T cells prevent catastrophic autoimmunity throughout the lifespan of mice. Nat Immunol 8: 191–197. 17136045 10.1038/ni1428

[7] Schildknecht A, Brauer S, Brenner C, Lahl K, Schild H, et al (2010) FoxP3+ regulatory T cells essentially contribute to peripheral CD8+ T-cell tolerance induced by steady-state dendritic cells. Proc Natl Acad Sci U S A 107: 199–203. 20018763 10.1073/pnas.0910620107 PMC2806715

[8] Muth S, Schütze K, Schild H, Probst HC (2012) Release of dendritic cells from cognate CD4+ T-cell recognition results in impaired peripheral tolerance and fatal cytotoxic T-cell mediated autoimmunity. Proc Natl Acad Sci U S A 109: 9059–9064. 22615402 10.1073/pnas.1110620109 PMC3384145

[9] Tang Q, Bluestone JA (2008) The Foxp3+ regulatory T cell: a jack of all trades, master of regulation. Nat Immunol 9: 239–244. 18285775 10.1038/ni1572 PMC3075612

[10] Vignali DA, Collison LW, Workman CJ (2008) How regulatory T cells work. Nat Rev Immunol 8: 523–532. 18566595 10.1038/nri2343 PMC2665249

[11] Chen ML, Pittet MJ, Gorelik L, Flavell RA, Weissleder R, et al (2005) Regulatory T cells suppress tumor-specific CD8 T cell cytotoxicity through TGF-beta signals in vivo. Proc Natl Acad Sci USA 102: 419–424. 15623559 10.1073/pnas.0408197102 PMC544311

[12] Mempel TR, Pittet MJ, Khazaie K, Weninger W, Weissleder R, et al (2006) Regulatory T cells reversibly suppress cytotoxic T cell function independent of effector differentiation. Immunity 25: 129–141. 16860762 10.1016/j.immuni.2006.04.015

[13] Toka FN, Suvas S, Rouse BT (2004) CD4+ CD25+ T cells regulate vaccine-generated primary and memory CD8+ T-cell responses against herpes simplex virus type 1. J Virol 78: 13082–13089. 15542660 10.1128/JVI.78.23.13082-13089.2004 PMC525021

[14] McNally A, Hill GR, Sparwasser T, Thomas R, Steptoe RJ (2011) CD4+CD25+ regulatory T cells control CD8+ T-cell effector differentiation by modulating IL-2 homeostasis. Proc Nat Acad Sci USA 108: 7529–7534. 21502514 10.1073/pnas.1103782108 PMC3088596

[15] Kastenmuller W, Gasteiger G, Subramanian N, Sparwasser T, Busch DH, et al (2011) Regulatory T cells selectively control CD8+ T cell effector pool size via IL-2 restriction. J Immunol 187: 3186–3197. 21849683 10.4049/jimmunol.1101649 PMC3169715

[16] Höfer T, Krichevsky O, Altan-Bonnet G (2012) Competition for IL-2 between Regulatory and Effector T Cells to Chisel Immune Responses. Front Immunol 3: 268. 22973270 10.3389/fimmu.2012.00268 PMC3433682

[17] Busse D, de la Rosa M, Hobiger K, Thurley K, Flossdorf M, et al (2010) Competing feedback loops shape IL-2 signaling between helper and regulatory T lymphocytes in cellular microenvironments. Proc Natl Acad Sci U S A 107: 3058–3063. 20133667 10.1073/pnas.0812851107 PMC2840293

[18] Hogquist KA, Jameson SC, Heath WR, Howard JL, Bevan MJ, et al (1994) T cell receptor antagonist peptides induce positive selection. Cell 76: 17–27. 8287475 10.1016/0092-8674(94)90169-4

[19] Steptoe RJ, Ritchie JM, Wilson NS, Villadangos JA, Lew AM, et al (2007) Cognate CD4+ help elicited by resting dendritic cells does not impair the induction of peripheral tolerance in CD8+ T cells. JI 178: 2094–2103. 10.4049/jimmunol.178.4.2094 17277113

[20] Steptoe RJ, Ritchie JM, Harrison LC (2002) Increased generation of dendritic cells from myeloid progenitors in autoimmune-prone non-obese diabetic mice. J Immunol 168: 5032–5041. 11994455 10.4049/jimmunol.168.10.5032

[21] Bertin-Maghit S, Pang D, O’Sullivan B, Best S, Duggan E, et al (2011) IL-1β produced in response to islet autoantigen presentation differentiates T-helper 17 cells at the expense of regulatory T cells: implications for the timing of tolerizing immunotherapy. Diabetes 60: 248–257. 20980463 10.2337/db10-0104 PMC3012178

[22] Steptoe RJ, Stankovic S, Lopaticki S, Jones LK, Harrison LC, et al (2004) Persistence of recipient lymphocytes in NOD mice after irradiation and bone marrow transplantation. JAI 22: 131–138. 10.1016/j.jaut.2003.12.003 14987741

[23] Shah JA, Darrah DA, Ambrozak DR, Turon TN, Mendez S, et al (2003) Dendritic cells are responsible for the capacity of CpG oligodeoxynucleotides to act as an adjuvant for potective vaccine immunity against Leishmania major in mice. J Exp Med 198: 281–291. 12874261 10.1084/jem.20030645 PMC2194077

[24] Drutman SB, Trombetta ES (2010) Dendritic cells continue to capture and present antigens after maturation in vivo. J Immunol 185 2140–2146. 10.4049/jimmunol.1000642 PMC2928255 20644175

[25] Grinberg-Bleyer Y, Saadoun D, Baeyens A, Billiard F, Goldstein JD, et al (2010) Pathogenic T cells have a paradoxical protective effect in murine autoimmune diabetes by boosting Tregs. J Clin Invest 120: 4558–4568. 21099113 10.1172/JCI42945 PMC2993590

[26] Sutmuller RP, van Duivenvoorde LM, van Elsas A, Schumacher TN, Wildenberg ME, et al (2001) Synergism of cytotoxic T lymphocyte-associated antigen 4 blockade and depletion of CD25(+) regulatory T cells in antitumor therapy reveals alternative pathways for suppression of autoreactive cytotoxic T lymphocyte responses. J Exp Med 194: 823–832. 11560997 10.1084/jem.194.6.823 PMC2195955

[27] Kornete M, Piccirillo CA (2012) Functional crosstalk between dendritic cells and Foxp3+ regulatory T cells in the maintenance of immune tolerance. Frontiers in Immunological Tolerance 3: Article 165. 10.3389/fimmu.2012.00165 PMC3381230 22737152

[28] Lahl K, Loddenkemper C, Drouin C, Freyer J, Arnason J, et al (2007) Selective depletion of Foxp3+ regulatory T cells induces a scurfy-like disease. J Exp Med 204: 57–63. 17200412 10.1084/jem.20061852 PMC2118432

[29] Serra P, Amrani A, Yamanouchi J, Han B, Thiessen S, et al (2003) CD40 ligation releases immature dendritic cells from the control of regulatory CD4+CD25+ T cells. Immunity 19: 877–889. 14670304 10.1016/s1074-7613(03)00327-3

[30] Ertelt JM, Buyukbasaran EZ, Jiang TT, Rowe JH, Xin L, et al (2013) B7–1/B7–2 blockade overrides the activation of protective CD8 T cells stimulated in the absence of Foxp3 regulatory T cells. J Leukoc Biol 94: 367–376. 23744647 10.1189/jlb.0313118 PMC3714566

[31] Liu H, Komai-Koma M, Xu D, Liew FY (2006) Toll-like receptor 2 signaling modulates the functions of CD4+ CD25+ regulatory T cells. Proc Natl Acad Sci USA 103: 7048–7053. 16632602 10.1073/pnas.0601554103 PMC1444884

[32] Pasare C, Medzhitov R (2003) Toll pathway-dependent blockade of CD4+CD25+ T cell-mediated suppression by dendritic cells. Science 299: 1033–1036. 12532024 10.1126/science.1078231

[33] Peng G, Guo Z, Kiniwa Y, Voo KS, Peng W, et al (2005) Toll-like receptor 8-mediated reversal of CD4+ regulatory T cell function. Science 309: 1380–1384. 16123302 10.1126/science.1113401

[34] Hsieh CS, Bautista JL (2010) Sliding set-points of immune responses for therapy of autoimmunity. J Exp Med 207: 1819–1823. 20805565 10.1084/jem.20101606 PMC2931165

[35] Boyman O, Sprent J (2012) The role of interleukin-2 during homeostasis and activation of the immune system. Nat Rev Immunol 17: 180–190. 10.1038/nri3156 22343569

[36] Malek TR (2008) The Biology of Interleukin-2. Annual Review of Immunology 26: 453–479. 10.1146/annurev.immunol.26.021607.090357 18062768

[37] Holz LE, Benseler V, Vo M, McGuffog C, Van Rooijen N, et al (2012) Naïve CD8 T cell activation by liver bone marrow-derived cells leads to a “neglected” IL-2low Bimhigh phenotype, poor CTL function and cell death. J Hepatol 57: 830–836. 22659099 10.1016/j.jhep.2012.05.015

[38] Hernandez J, Aung S, Redmond WL, Sherman LA (2001) Phenotypic and functional analysis of CD8(+) T cells undergoing peripheral deletion in response to cross-presentation of self-antigen. J Exp Med 194: 707–717. 11560988 10.1084/jem.194.6.707 PMC2195957

[39] Cheng LE, Greenberg PD (2002) Selective delivery of augmented IL-2 receptor signals to responding CD8+ T cells increases the size of the acute antiviral response and of the resulting memory T cell pool. J Immunol 169: 4990–4997. 12391213 10.4049/jimmunol.169.9.4990

[40] Setoguchi R, Hori S, Takahashi T, Sakaguchi S (2005) Homeostatic maintenance of natural Foxp3(+) CD25(+) CD4(+) regulatory T cells by interleukin (IL)-2 and induction of autoimmune disease by IL-2 neutralization. J Exp Med 201: 723–735. 15753206 10.1084/jem.20041982 PMC2212841

[41] Almeida AR, Zaragoza B, Freitas AA (2006) Indexation as a novel mechanism of lymphocyte homeostasis: the number of CD4+CD25+ regulatory T cells is indexed to the number of IL-2-producing cells. J Immunol 177: 192–200. 16785514 10.4049/jimmunol.177.1.192

